# Genetic requirements for uropathogenic *E. coli* proliferation in the bladder cell infection cycle

**DOI:** 10.1128/msystems.00387-24

**Published:** 2024-09-17

**Authors:** Daniel G. Mediati, Tamika A. Blair, Ariana Costas, Leigh G. Monahan, Bill Söderström, Ian G. Charles, Iain G. Duggin

**Affiliations:** 1 Australian Institute for Microbiology and Infection, University of Technology Sydney, Ultimo, Australia; 2 Institut Cochin, INSERM U1016, Université de Paris, Paris, France; 3 Quadram Institute Bioscience, Norwich Research Park, Norwich, United Kingdom; 4 Norwich Medical School, University of East Anglia, Norwich Research Park, Norwich, United Kingdom; Chan Zuckerberg Biohub, Stanford, California, USA

**Keywords:** UTI, UPEC, TraDIS, cystitis, stage-resolved model, intracellular infection

## Abstract

Uropathogenic *Escherichia coli* (UPEC) requires an adaptable physiology to survive the wide range of environments experienced in the host, including gut and urinary tract surfaces. To identify UPEC genes required during intracellular infection, we developed a transposon-directed insertion-site sequencing approach for cellular infection models and searched for genes in a library of ~20,000 UTI89 transposon-insertion mutants that are specifically required at the distinct stages of infection of cultured bladder epithelial cells. Some of the bacterial functional requirements apparent in host bladder cell growth overlapped with those for M9-glycerol, notably nutrient utilization, polysaccharide and macromolecule precursor biosynthesis, and cell envelope stress tolerance. Two genes implicated in the intracellular bladder cell infection stage were confirmed through independent gene deletion studies: *neuC* (sialic acid capsule biosynthesis) and *hisF* (histidine biosynthesis). Distinct sets of UPEC genes were also implicated in bacterial dispersal, where UPEC erupts from bladder cells in highly filamentous or motile forms upon exposure to human urine, and during recovery from infection in a rich medium. We confirm that the *dedD* gene linked to septal peptidoglycan remodeling is required during UPEC dispersal from human bladder cells and may help stabilize cell division or the cell wall during envelope stress created by host cells. Our findings support a view that the host intracellular environment and infection cycle are multi-nutrient limited and create stress that demands an array of biosynthetic, cell envelope integrity, and biofilm-related functions of UPEC.

**IMPORTANCE:**

Urinary tract infections (UTIs) are one of the most frequent infections worldwide. Uropathogenic *Escherichia coli* (UPEC), which accounts for ~80% of UTIs, must rapidly adapt to highly variable host environments, such as the gut, bladder sub-surface, and urine. In this study, we searched for UPEC genes required for bacterial growth and survival throughout the cellular infection cycle. Genes required for *de novo* synthesis of biomolecules and cell envelope integrity appeared to be important, and other genes were also implicated in bacterial dispersal and recovery from infection of cultured bladder cells. With further studies of individual gene function, their potential as therapeutic targets may be realized. This study expands knowledge of the UTI cycle and establishes an approach to genome-wide functional analyses of stage-resolved microbial infections.

## INTRODUCTION

Microbes are frequently challenged by highly variable environments and limited supplies of essential nutrients. An important example of the need to flexibly adapt to such dynamic conditions is provided by uropathogenic *Escherichia coli* (UPEC), which originate from the gut but have adapted the ability to disseminate and colonize the urinary tract. In an acute urinary tract infection (UTI), UPEC cells undergo an intracellular infection cycle that was first defined in infected mouse bladder explants using time-lapse microscopy (1). Briefly, UPEC adheres to the luminal surface of the bladder epithelium and becomes internalized (2). Once in the host cell cytoplasm, UPEC proliferates and forms biofilm-like intracellular bacterial communities (IBCs) (3). When an IBC reaches an advanced state (up to 10^5^ bacteria per host cell), the IBC switches to a dispersal mode, accompanied by host bladder cell rupture and the release of subpopulations of highly filamentous or motile UPEC (1, 4). Released UPEC filaments are viable (4, 5), and filamentation is reversible (6, 7), which allows re-invasion of adjacent bladder cells (8). The diverse range of environments UPEC experience in the gut, urinary tract epithelium, and urine—often involving rapid nutrient transitions—underscores the importance of a flexible metabolic capacity (9).

Genome-wide approaches to identify genes encoding the transporters, regulators, and metabolic enzymes required for growth under various culture conditions provide a way to understand the metabolic capacity and flexibility needed for infection and may lead to the identification of potential targets for antimicrobial therapies. Early studies assessed *E. coli* gene essentiality under rich and minimal nutrient media by screening collections (libraries) of transposon-insertion or knock-out mutant strains (10, 11). More recently, techniques for mapping transposon insertion libraries using high-throughput DNA sequencing, collectively known as transposon insertion-site sequencing (TIS), have expanded the capacity for identifying essential genes (12–14). The approach has also been developed to identify conditionally essential genes, such as after exposure to serum (15), bacteriophage (16), urine (17), and antimicrobials (18–20), or through various physical selection methods that define cellular phenotypes or developmental pathways (12, 21).

Similar approaches to identify genes required for infection have been limited by the moderate scales associated with many experimental infection models, combined with the large number of unique bacterial mutants that would need to be simultaneously screened with TIS (12, 22). Several studies have used TIS to map pathogen genes important for infection in suitable animal models (23–27). Transposon-directed insertion-site sequencing (TraDIS) was first applied to UPEC to identify the gene profile required for *in vitro* growth of EC958 (an *E. coli* ST131 clonal lineage) in human serum (15). Recently, UPEC gene requirements in acute UTI were assessed using pig and diuretic-mouse models to help overcome the limited scale of the standard mouse model of cystitis (28, 29). The results suggested that many bacterial genes, especially those involved in certain nutrient uptake and lipopolysaccharide (LPS) biosynthesis pathways, are important in UTI.

Here, we sought to identify genes specifically required during distinct stages of the intracellular infection cycle by using a stage-resolved human bladder cell culture model of infection (4, 8, 30). We first modified the TraDIS protocol to streamline sequencing, with less input DNA required than the original protocol (14), and applied this to a moderate-scale transposon-insertion library suitable for infection models, consisting of ~20,000 unique mutants in the model UPEC cystitis isolate UTI89. We used TraDIS to map transposon-insertion sites in the genome of UTI89 harvested from a pre-infection inoculum and following 24 h intracellular bladder cell growth. Surprisingly, we find that many metabolic genes with reduced survival in cultured human bladder cells are also important for growth passaging in minimal M9 media, confirming that many metabolic pathways rely on *de novo* synthesis during intracellular bladder infection. We find that deletions of the histidine biosynthesis gene *hisF* and *neuC* involved in the biosynthesis of cell surface capsule are attenuated after 24 h growth in cultured human bladder cells. By mapping UPEC gene importance at the distinct later stages of cellular infection, we have also generated a dataset of genes implicated in bacterial dispersal from host cells and recovery in media. We further demonstrate that the SPOR domain-containing protein DedD involved in peptidoglycan remodeling is required during UPEC dispersal from bladder cells and likely plays a role in stabilizing the bacterial envelope during host cell stress in the latter stages of the infection cycle. This work implicates genetic targets for further analyzes of the bladder cell infection cycle and for potential therapeutic intervention in UTI.

## RESULTS

### Construction of a transposon mutant library in *E. coli* UTI89 and its characterization by a modified TraDIS protocol

**Fig 1:**
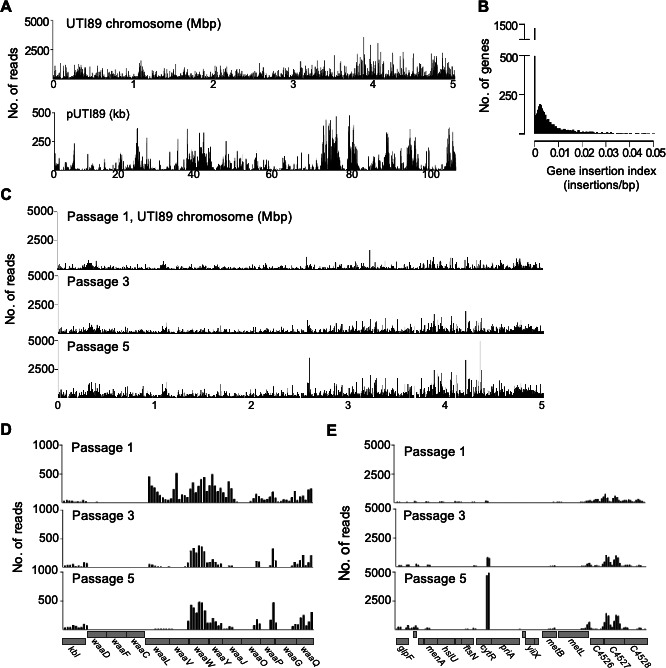
TraDIS mapping of an *E. coli* UTI89 transposon-insertion library. (**A**) The distribution of TraDIS mapped-reads in 150 bp windows along the ~5.06 Mbp UTI89 chromosome and ~114 kb plasmid, pUTI89. (**B**) Frequency distribution of the gene insertion index—the number of Tn-insertions within each gene divided by that gene’s length (in bp)—in the UTI89 Tn-insertion library. (**C**) The number of mapped-reads in 150 bp windows along the UTI89 chromosome following daily passaging (1/400) in LB media. Data were combined from both Tn-ends to generate these plots. (**D**) Examples of the read-count distribution (150 bp windows) from one culture replicate, encompassing 4,053,040–4,066,540 bp, showing a decrease in abundance of insertion mutants in *waaV* and *waaL*, and (**E**) 4,394,185–4,413,535 bp, showing an increase in abundance of insertion mutants in *cytR* over time.

Transposon mutagenesis of UTI89 was done by electroporation of the mini-Tn5 synthetic transposon, Ez-Tn5<Kan-2>, assembled with modified Tn5 transposase (“transposasomes”). Electroporations yielded 97,500 kanamycin-resistant colony-forming units (cfu). Colonies were resuspended, pooled, and then stored in frozen aliquots (OD_600nm_ = 10), which upon thawing contained 1.18 × 10^10^ cfu/mL. We developed a modified TraDIS protocol by initially fragmenting total DNA purified from the library with tagmentation, which requires less input DNA than the original method and is likely advantageous in host cell infection models where bacterial yields are limited (Fig. S1A). The transposon-genome junctions were amplified from both ends of the transposon separately (Fig. S1B) so that standard Illumina sequencing could be used to identify the insertion sites while avoiding the need for custom primers or dark sequencing cycles (Fig. S1C). Sequence data analyzes identified an average of 18,756 unique transposon-insertion sites in the chromosome and 464 in the endogenous plasmid, pUTI89 (low copy number plasmid, 1–2 copies per cell). Transposon-insertions were distributed throughout the genome, equating to an average transposon-insertion frequency of one per 270 bp in the chromosome and slightly higher in pUTI89 (Fig. 1A; Table S1A).

A strong correlation (*R*^2^ = 0.878) was observed for the number of transposon-insertion sites identified within each annotated gene obtained from the two transposon-ends (Fig. S2). Approximately 22% of genes (~1,200) did not have a detected transposon-insertion (i.e., they had an insertion index of zero, Fig. 1B), and they are listed in Table S1B. It is predicted that 8%–10% of an *E. coli* genome contains genes that are individually essential for growth in LB medium (13, 31). The mean length of transposon-free open reading frames was 569 bp compared to the genome-wide mean of 901 bp, consistent with a minor proportion of non-essential genes containing no transposon-insertion. Our aim was to avoid experimental bottlenecks, that is random loss of mutants caused by the insufficient feasible scale of infection models. Genes containing at least one transposon insertion gave rise to a symmetrical frequency distribution of the pooled gene insertion index (insertions/gene/bp; Fig. 1B), consistent with an approximately near-random insertion frequency (14, 32).

Prior to comparing the UTI89 transposon library in various selective conditions, we first analyzed the relative abundance of mutants in the library over time by passaging the library in LB once per day over 5 days. Cultures harvested after days 1, 3, and 5 were analyzed by TraDIS (Fig. 1C). Genome-wide analysis identified 196 genes with a reduction in read counts from days 1 to 5 [log fold-change (FC) ≤ −2 and *p* ≤ 0.05], suggesting that disruption of these genes reduced the growth rate in LB (Table S1C; e.g., *waaL* encoding the O-antigen ligase; Fig. 1D). Conversely, 16 genes showed a substantial increase in the relative abundance of transposon-insertion reads by day 5 (e.g., the cytidine transcriptional repressor *cytR*; Fig. 1E). These results reflect the dynamic behavior of transposon mutant libraries and may serve as a reference for comparing changes seen in other conditions of long-term growth to improve the specificity of identifying conditionally essential genes.

### UTI89 gene requirements during infection of cultured human bladder epithelial cells

Stages of the UPEC intracellular infection cycle were first demonstrated in mouse bladder explants (1). However, much remains unknown about the UPEC genetic requirements during each distinct stage of the acute infection cycle. One of the challenges is that the stages of cellular infection that are resolvable by microscopy are asynchronous at the organ level and are not readily resolved by high-throughput library screens on current *in vivo* models. Furthermore, the limited scale of standard *in vivo* infection models can severely limit the simultaneous screening of large libraries of mutants (a term called “bottlenecking”).

**Fig 2:**
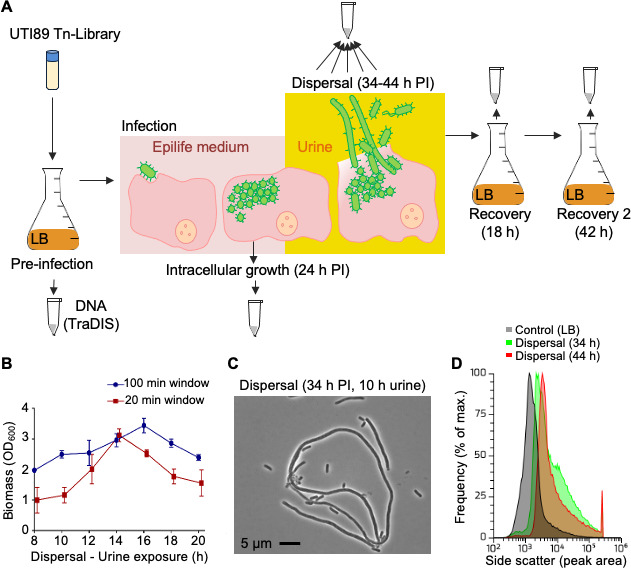
Cell culture model of UTI89 infection of BECs for TraDIS analysis. (**A**) The stages of infection and the pre- and post-infection samples that were subsequently analyzed by TraDIS are represented by sample tubes. PI = post-infection. (**B**) Optical density measurements (OD_600nm_) of samples harvested in 20- or 100-min windows during the urine dispersal phase in the infection model. Error bars indicate the SD. (**C**) Phase-contrast micrograph of a dispersion sample shows a mixture of rod-shaped and highly filamentous UPEC. (**D**) Flow cytometry frequency distributions of cell size (represented by the side-scatter peak area), showing populations of high-scatter filamentous cells during dispersal.

**Fig 3:**
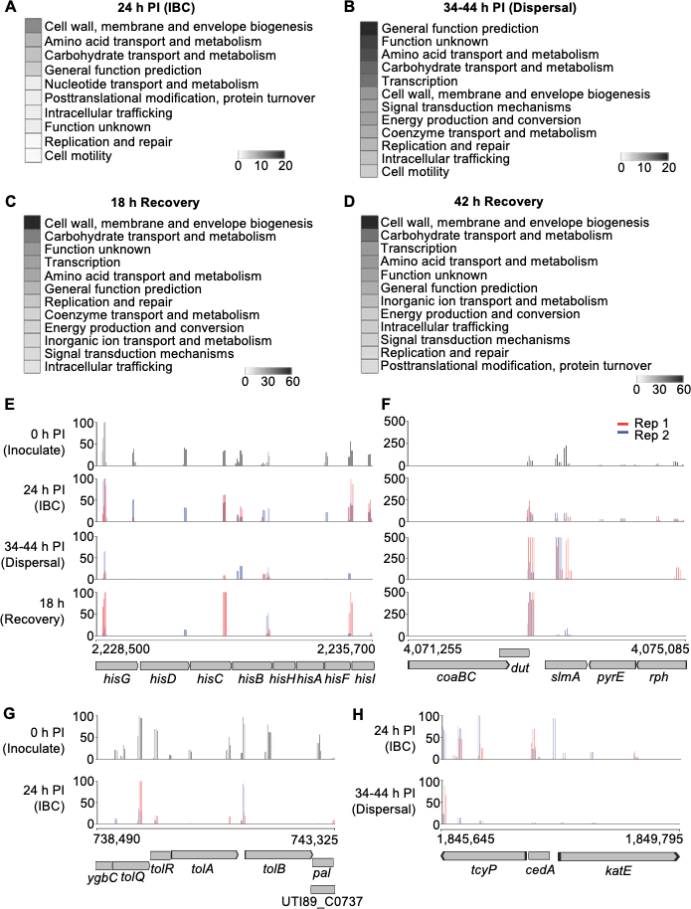
TraDIS read counts within selected UTI89 genomic loci at the indicated stages of BEC infection and recovery. (**A–D**) COGs detailed for statistically significant genes found within the TraDIS datasets in all stages of the infection and recovery process (adjusted *p* < 0.05). (**E and F**) Mapped read counts in the indicated genomic regions in all stages of the infection and recovery process that were sampled by TraDIS. The two experimental replicates are shown with blue and red bars. (**G**) Mapped read counts from the Inoculate (top panel) and IBC (bottom panel) samples encompassing the indicated *tol* and *pal* genes. (**H**) Mapped read counts from the IBC (top panel) and Dispersal (bottom panel) samples encompassing genes from the *cedA* region. Each plot has a bin size of 50 bp.

An established *in vitro* human bladder epithelial cell (BEC) infection model has allowed bacterial sampling at the distinct stages of the intracellular infection cycle (8) and recapitulates the stages previously observed by microscopy *in vivo* (1, 33). To assess UPEC intracellular growth within BECs, we performed TraDIS using an up-scaled model of the established *in vitro* human BEC infection (8) that enabled high yields of bacteria to be harvested at specific stages of intracellular infection (Fig. 2A). We first grew the UTI89 transposon library statically in LB, then took a pre-infection (or “inoculate”) sample for TraDIS, and we infected three 100 mm culture dishes, in biological duplicate (*n* = 6), containing confluent BECs for each infection stage to be sampled (multiplicity of infection, MOI = 100). UTI89 surviving intracellular infection were harvested at 24 h post-infection (IBC/intracellular growth; Fig. 2A). Dense bacterial aggregates within our *in vitro* BEC model at 18 h and 22 h post-infection resembled previously identified IBCs (1, 33) (Fig. S3). Bacteria surviving 34–44 h post-infection were harvested in 20 min windows of concentrated urine replenishment (density > 1.025 g/mL) and demonstrated high bacterial yields (extracellular host cell dispersal; Fig. 2B). Concentrated urine induces a sub-population of UPEC as filaments (4), and we observed characteristic filamentous UPEC obtained from within the dispersal stage of UTI within our *in vitro* BEC infection model (Fig. 2C and D). Extracellular bacteria from the dispersal sample were resuspended in LB and harvested after 18 h recovery, and a sample of this was subjected to a further 24 h passage in LB (total 42 h; Fig. 2A). Samples from these distinct stages were collected for TraDIS analyses (read count statistics are shown in Table S1A, and each gene’s transposon-specific counts are demonstrated in Table S1D). The complete TraDIS analysis output is given in Table S2. We applied stringent criteria (*p* ≤ 0.05 and logFC ≤ −1.0) to each TraDIS sample dataset to improve the accuracy of gene identification. Clusters of orthologous group (COG) analyses for each UTI89 TraDIS sample dataset and the distribution of reads for select loci are shown in Fig. 3. Certain mutants were selectively lost at different stages of infection, as would be expected for genes important for that corresponding stage of infection.

### UTI89 genes implicated in intracellular growth of cultured human BECs (IBC stage)

To identify genes required for intracellular infection, the TraDIS read counts for each mutant obtained from the 24 h IBC samples were compared to those from the pre-infection sample (Table S2A). A total of 37 genes met the stringent criteria in both replicates (Table 1). Some of these genes are related to carbon metabolism, precursor biosynthesis, and cell size control (e.g., *galU*), amino-acid biosynthesis and transport (e.g., *aroK*), and iron acquisition (e.g., *iroE*), which is consistent with a reliance on *de novo* biosynthesis of some essential metabolites, amino acids, and sugars during intracellular infection.

**TABLE 1:** UTI89 genes important for 24 h intracellular BEC growth

Locus ID	Gene^*a*^	Annotation and function	LogFC^*b*^	*p*-value^*b*^	*q*-value^*b*^	Implicated in UTI pathogenesis^*c*^
UTI89_C4063	*yhjM*	(*bcsZ*) endo-1,4-D-glucanase (extracellular cellulose biosynthesis)	−8.85	7.92E-04	7.13E-02	This study
UTI89_C0216	*yaeD*	(*gmhB*) ADP-heptose biosynthesis, outer membrane LPS biosynthesis	−8.13	4.13E-03	1.64E-01	This study
UTI89_C1432	*galU*	UDP-glucose biosynthesis (polysaccharide, LPS Lipid A-core, etc. biosynthesis)	−8.03	2.74E-04	5.78E-02	(34, 35)
UTI89_C2859		Hypothetical protein	−7.53	6.71E-03	2.10E-01	This study
UTI89_C3001	*ymfM*	Prophage cell division inhibitor	−7.26	1.00E-02	2.66E-01	This study
UTI89_C1022	*ompA*	Outer membrane protein A (outer membrane stability and biofilm formation)	−6.85	2.09E-07	2.32E-04	(36)
UTI89_C4372	*dapF*	Diaminopimelate epimerase (lysine/diaminopimelate biosynthesis)	−6.79	9.62E-04	7.78E-02	This study
UTI89_C2145	*fliM*	Flagellar motor switch complex protein	−6.65	2.43E-03	1.28E-01	This study
UTI89_C1677		M35 metalopeptidase-like protein	−6.42	8.21E-04	8.96E-02	This study
UTI89_C3888	*aroK*	Shikimate kinase I (aromatic amino acid biosynthesis)	−6.36	4.85E-03	1.51E-01	This study
UTI89_C2018	*yobD*	Inner membrane protein (uncharacterized)	−6.22	5.42E-03	1.92E-01	This study
UTI89_C1392	*dhaL*	(*ycgS*) dihydroxyacetone kinase (glycerol assimilation)	−5.96	2.39E-02	3.97E-01	This study
UTI89_C0775	*bioC*	Methyltransferase (biotin biosynthesis)	−5.92	3.27E-03	1.52E-01	This study
UTI89_C0507	*fsr*	Efflux pump (drug resistance)	−5.86	6.33E-03	2.02E-01	This study
UTI89_C4165	*waaF*	(*rfaF*) ADP-heptose LPS heptosyltransferase II (LPS core biosynthesis)	−5.83	3.52E-03	1.57E-01	(28, 37)
UTI89_C0736	*tolB*	Periplasmic Tol-Pal system protein (cell envelope function and integrity)	−5.75	7.03E-04	8.42E-02	(28, 38)
UTI89_C3378	*gspK*	Inner membrane pseudopilin (type II secretion)	−5.67	3.80E-03	1.59E-01	This study
UTI89_C0552		Hypothetical protein	−5.51	4.48E-03	1.50E-01	This study
UTI89_C0693	*pgm*	Phosphoglucomutase (UDP-glucose, polysaccharide, LPS Lipid A-core, etc. biosynthesis)	−5.37	2.38E-05	7.62E-03	(39)
UTI89_C1951	*ynjC*	Inner membrane ABC transporter permease	−5.28	5.02E-03	1.75E-01	This study
UTI89_C4231	*yidG*	Inner membrane protein (uncharacterized)	−5.21	2.07E-02	2.90E-01	This study
UTI89_C2768	*amiA*	Periplasmic N-acetylmuramoyl-L-alanine amidase (peptidoglycan cleavage)	−5.16	2.78E-02	4.59E-01	(40)
UTI89_C0934		Putative phage tail protein	−4.97	2.67E-02	3.48E-01	This study
UTI89_C0892	*csy1*	Types I–F CRISPR-associated protein	−4.93	1.36E-03	9.86E-02	This study
UTI89_C1393	*dhaK*	(*ycgT*) dihydroxyacetone kinase (glycerol assimilation)	−4.57	2.40E-02	3.92E-01	This study
UTI89_C2031	*prc*	Carboxy-terminal protease (multiple effects on stress tolerance and envelope integrity)	−4.28	5.80E-03	1.69E-01	(28, 41)
UTI89_C0181	*glnD*	(nitrogen regulatory protein-PII) uridylyltransferase	−3.80	2.07E-02	3.71E-01	This study
UTI89_C2954	*smpB*	SsrA-binding protein (degradation of stalled translation polypeptides)	−3.69	2.66E-02	4.47E-01	This study
UTI89_C2430	*lysP*	Lysine-specific uptake transporter	−3.60	2.85E-02	4.48E-01	(42)
UTI89_C0235	*aspV*	Aspartate tRNA	−3.28	2.80E-02	4.59E-01	(43)
UTI89_C2119	*yecS*	Cystine ABC transporter integral membrane subunit	−2.78	3.24E-02	4.86E-01	This study
UTI89_C3724	*yrdA*	γ-Class carbonic anhydrase (pH regulation)	−2.78	3.11E-03	1.35E-01	This study
UTI89_C0070		Hypothetical protein	−2.57	1.68E-02	3.25E-01	This study
UTI89_C3503	*dnaG*	DNA primase (essential DNA replication protein)	−2.41	1.40E-02	3.17E-01	This study
UTI89_C3036	*nrdF*	Ribonucleoside-diphosphate reductase 2 beta-chain (pyrimidine biosynthesis)	−2.33	3.72E-05	7.32E-03	(44)
UTI89_C1119	*iroE*	Catecholate siderophore esterase (iron utilization)	−2.13	2.35E-02	3.56E-01	This study
UTI89_C0136	*cueO*	Periplasmic multicopper oxidase	−2.03	2.42E-03	1.07E-01	This study

^*a*^ Genes encoding hypothetical proteins, or those with no characterized homologs, are left blank. Most predicted functions have been assigned with reference to *E. coli* K-12 gene function.

^*b*^ LogFC and significance scores (*p*-value and *q*-value) are shown as the means from the two independently analyzed replicates. The *q*-value is the corrected *p*-value or false discovery probability.

^*c*^ Previously implicated in pathogenesis of acute UTI either through single gene knockout experiments or high-throughput genetic screening.

### UTI89 genes implicated in UPEC dispersal from infection of BECs

When an IBC reaches an advanced state, conditions that remain unresolved trigger bacterial dispersal from host cells (1, 8). UPEC may differentiate into highly filamentous (cell division arrest) or motile forms at this stage to aid dispersal (4, 45). To identify genes involved in progression to this poorly understood phase, we obtained extracellular UTI89 transposon-insertion mutants released from the bladder cells by collecting the supernatant between 34-44 h post-infection (post-gentamicin treatment and during exposure to human urine of density > 1.025 g/mL, which induce host cell dispersal) (4). The resulting TraDIS data were analyzed by comparison to the IBC stage to identify genes specifically important for dispersal; the complete analysis output is given in Table S2B, and a total of 151 genes met the stringent criteria in both replicates (Table S2C). Some of these genes have been previously implicated in the regulation of cell division, including *cedA* (46, 47), *ytfB* (48), *zwf* (7)*,* and *ftsX* [recently reviewed in reference (49)], and cell wall remodeling such as *dacA* (PBP5) (50) and *dedD* (51) (Table S2C). Notably, while the *waaL* gene involved in LPS biosynthesis also appeared in this list, *waaL* mutants diminished rapidly over time in our library even in LB (Fig. 1).

### UPEC genes implicated in bacterial recovery from infection of BECs

Upon dispersal, filamentous UPEC can revert to rod cells through unknown mechanisms (5, 8). This is thought to be necessary for long-term bacterial survival and for regaining invasive capacity to initiate a new cycle of infection. To search for genes important for bacterial recovery after dispersal, bacteria harvested from the dispersal phase were collected and then resuspended in LB and allowed to grow for 18 h (recovery sample). A sample of this culture was then grown for a further 24 h in liquid LB (42 h recovery sample; Fig. 2A). TraDIS data obtained from these recovery samples were then separately compared to the dispersal TraDIS dataset to identify any mutants that had a significantly reduced ability to recover from infection (Tables S2D and S2E). A total of 306 and 318 genes met the stringent criteria at the 18 h and 42 h recovery timepoints, respectively (Table S2F and S2G). A total of 233 genes were found in common between the recovery samples, and 40 chromosomal genes were found specific to the 18 h recovery sample, which included some previously implicated in the regulation of cell division, the cell envelope stress response, and peptidoglycan remodeling, including *yfiR* (52), *yibP* (*envC*) (53), and *ampH* (54), consistent with likely requirements for bacterial recovery from envelope stresses experienced during intracellular infection (5). For example, YfiR is known to promote UPEC survival in the mouse model of UTI (55), supporting our TraDIS analyses in the identification of *bona fide* genetic requirements for bladder cell infection.

### Identification of genes important for UTI89 growth in M9-glycerol

The metabolic capacity of UPEC is highly responsive to fluctuating nutrient availability. UPEC must adapt to new nutritional standings, which is evident in the transition from the intestine to the urinary tract. Considering the different nutrient availabilities, metabolites within the urine and nutrient-limited bladder cells are most likely to activate metabolic adaptive strategies in UPEC to survive (9).

**Fig 4:**
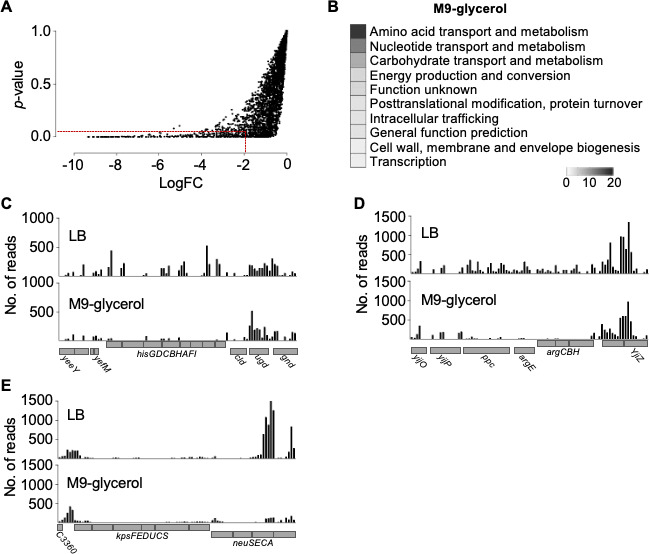
Identification of genes important for growth in M9-glycerol compared to LB. (**A**) Plot of each gene’s *p*-value against its corresponding logFC value, resulting from the comparison of transposon-insertion counts in M9-glycerol and LB media. The dashed lines indicate the stringent threshold criteria (*p* ≤ 0.05 and logFC < −2). (**B**) COGs detailed for the 42 statistically significant genes found to be required for growth in M9-glycerol (adjusted *p* < 0.05). (**C–E**) Representative read counts from one biological replicate at selected loci (read counts determined in 150 bp windows). The position of the annotated gene is indicated (*below*). (**B**) Coordinates 2,225,678–2,239,628 bp. (**C**) Coordinates 4,431,758–4,445,258 bp. (**D**) Coordinates 3,288,114–3,301,914 bp.

To identify genes that become important for growth or survival during a transition to nutrient-limited conditions, approximating nutritional changes that bacteria may experience when moving between various host environments, we grew the transposon-insertion library in LB to early-log phase and then diluted 1/10 into M9-glycerol minimal medium and LB (control), in duplicate, and sampled the four cultures after three 24 h passages for TraDIS analyses. Read counts and basic statistics for the dataset are in Table S1A, and the complete output from analyses comparing individual genes between M9-glycerol and LB is in Table S1E. A total of 42 stringent genes were implicated as being important for growth in M9-glycerol (Fig. 4A; Table 2). Many of these significantly underrepresented mutants after the transition to M9-glycerol are implicated in catabolic functions (Fig. 4B), and many overlap with COG functional classes from the intracellular stage (IBC) of infection, including “amino acid transport and metabolism,” “carbohydrate transport and metabolism,” and “cell wall, membrane, and envelope biogenesis” (Fig. 3A).

**TABLE 2:** UTI89 genes important for growth in M9-glycerol, compared to LB

Locus ID	Gene^a^	Likely function	LogFC^b^	*p*-value^b^	*q*-value^b^
UTI89_C0551	*purE*	Ribosylaminoimidazole carboxylase (purine biosynthesis)	−8.21	4.37E-04	2.12E-02
UTI89_C2298	*hisF*	Imidazole glycerol phosphate synthase (histidine biosynthesis)	−7.29	4.26E-04	2.06E-02
UTI89_C1010	*pyrD*	Dihydroorotate dehydrogenase (pyrimidine biosynthesis)	−6.72	1.74E-02	1.63E-01
UTI89_C3926	*glpD*	Glycerol-3-phosphate dehydrogenase (aerobic growth on glycerol)	−6.57	5.53E-04	1.91E-02
UTI89_C0736	*tolB*	Periplasmic Tol-Pal system protein (cell envelope function and integrity)	−6.00	9.30E-03	1.01E-01
UTI89_C3111	*rpoS*	Stress response RNA pol sigma factor (expression of genes for metabolism in minimal medium/urine)	−5.95	3.58E-03	6.66E-02
UTI89_C3110		Hypothetical protein overlaps with *rpoS* (opposite strand; may reflect role of *rpoS* here)	−5.94	3.66E-03	6.77E-02
UTI89_C0737		Hypothetical protein overlaps with *tolB* and *pal* (opposite strand; may reflect role of Tol-Pal here)	−5.90	1.93E-02	1.12E-01
UTI89_C1127		Putative IS4 family transposase	−5.83	6.98E-04	2.53E-02
UTI89_C3887	*aroB*	3-Dehydroquinate synthase (aromatic amino acid biosynthesis)	−5.66	4.12E-03	7.37E-02
UTI89_C2448	*yeiP*	Putative elongation factor uncharacterized (regulated by cAMP/CRP that controls catabolic genes)	−5.64	1.92E-03	3.81E-02
UTI89_C3726	*aroE*	Shikimate 5-dehydrogenase (aromatic amino acid biosynthesis)	−5.62	1.20E-02	9.33E-02
UTI89_C3429	*yghB*	DedA family protein (possible role in inner membrane integrity during stress)	−5.55	7.78E-03	8.00E-02
UTI89_C0733	*tolQ*	Inner membrane Tol-Pal system protein (cell envelope function and integrity)	−5.49	1.51E-02	1.06E-01
UTI89_C4186	*pyrE*	Rotate phosphoribosyltransferase (pyrimidine biosynthesis)	−5.44	1.31E-02	1.50E-01
UTI89_C3121	*cysC*	Adenylyl-sulfate kinase (sulfate assimilation)	−5.38	7.30E-03	7.32E-02
UTI89_C2889		Conserved hypothetical protein (unknown function)	−5.19	1.11E-02	1.05E-01
UTI89_C4551	*argH*	Arginine-succinate lyase (arginine biosynthesis)	−5.01	1.21E-04	6.01E-03
UTI89_C4510	*glpK*	Aerobic glycerol 3-phosphate dehydrogenase (required for growth on glycerol)	−4.98	6.55E-09	1.02E-06
UTI89_C3219	*argA*	N-acetylglutamate synthase (arginine/ornithine biosynthesis)	−4.81	1.24E-05	7.18E-04
UTI89_C3814	*purH*	Formyltransferase/IMP cyclohydrolase (purine biosynthesis)	−4.80	3.29E-03	6.53E-02
UTI89_C1621		Hypothetical protein (DUF333-containing protein)	−4.49	4.81E-03	8.12E-02
UTI89_C1620		Hypothetical protein	−4.21	9.48E-03	1.26E-01
UTI89_C0037	*carB*	Carbamoyl-phosphate synthase (arginine/pyrimidine biosynthesis)	−4.20	3.86E-05	4.38E-03
UTI89_C2877	*purL*	Phosphoribosylformylglycinamide synthetase (purine biosynthesis)	−4.19	2.72E-04	1.66E-02
UTI89_C0465	*clpP*	Serine protease (multiple functions and survival during nutrient limitation)	−4.14	2.06E-02	1.96E-01
UTI89_C5159	*serB*	Phosphoserine phosphatase (serine biosynthesis)	−4.07	1.87E-02	1.65E-01
UTI89_C2296	*hisH*	Imidazole glycerol phosphate synthase (acts with HisF in histidine biosynthesis)	−3.94	2.23E-03	5.04E-02
UTI89_C0049	*fixC*	Xidoreductase (regulated by cAMP/CRP that controls catabolic genes, L-carnitine metabolism)	−3.54	8.81E-03	9.40E-02
UTI89_C3096	*ygbA*	Conserved hypothetical protein	−3.48	2.45E-02	1.85E-01
UTI89_C2933	*tyrA*	Chorismate mutase/prephenate (aromatic amino acid biosynthesis)	−3.29	5.97E-03	4.79E-02
UTI89_C1105		Hypothetical protein	−3.28	2.63E-02	2.01E-01
UTI89_C0231	*gloB*	Hydroxyacylglutathione hydrolase (glycerol assimilation via methylglyoxyl)	−3.27	2.67E-02	2.25E-01
UTI89_C2747	*cysK*	O-acetylserine sulfhydrolase A (cysteine biosynthesis)	−3.23	1.62E-02	1.58E-01
UTI89_C5098		Putative antirepressor	−2.95	3.11E-03	5.96E-02
UTI89_C4549	*argC*	N-acetyl-gamma-glutamyl-phosphate (ornithine/arginine biosynthesis)	−2.93	1.48E-02	1.58E-01
UTI89_C0888	*clpA*	Protease ATP-binding subunit (functions with *clpP*)	−2.83	3.14E-02	2.16E-01
UTI89_C3077	*ascF*	Phosphotransferase enzyme II (group translocation)	−2.82	1.46E-02	1.58E-01
UTI89_C4548	*argE*	Acetylornithine deacetylase (ornithine/arginine biosynthesis)	−2.32	2.91E-03	5.37E-02
UTI89_C3370	*neuC*	Sialic acid biosynthesis (cell surface capsule biosynthesis)	−2.20	7.64E-04	2.61E-02
UTI89_C0118	*ampD*	1,6-Anhydro-*N*-acetylmuramoyl-L-alanine amidase (peptidoglycan recycling)	−2.17	4.27E-03	7.63E-02
UTI89_C4223	*uhpC*	Inner membrane protein sensing glucose-6-phosphate (transport regulation)	−2.16	3.17E-02	2.51E-01

^a^ Genes encoding hypothetical proteins, or those with no characterized homologs, are left blank. Most predicted functions have been assigned with reference to *E. coli* K-12 gene function.

^b^ LogFC and significance scores (*p*-value and *q*-value) are shown as the means from the two independently analyzed replicates. The *q*-value is the corrected *p*-value or false discovery probability.

TraDIS data for three selected genomic regions containing significant differences between M9-glycerol and LB are shown in Fig. 4C through E. These included the histidine biosynthesis operon (Fig. 4C) and several genes for arginine biosynthesis (Fig. 4D). These genes are required during nutrient-limited conditions in *E. coli* K-12, and many of the other genes important in UTI89 (Table 2) also have known roles in catabolic functions in M9-glycerol cultures of the commensal model strain K-12 (11). A surprising example is *neuC* (Fig. 4E), encoding an epimerase for sialic acid biosynthesis, expected to be involved in cell surface polysialic acid production. Interestingly, *neuC* and the gene cluster it sits within for capsule production are not present in many other strains of *E. coli* that can grow well in M9-glycerol (e.g., K-12), consistent with the concept that gene requirements are strain and context dependent (56).

### Validation of select mutants for growth in M9-glycerol analyzed by TraDIS

**Fig 5:**
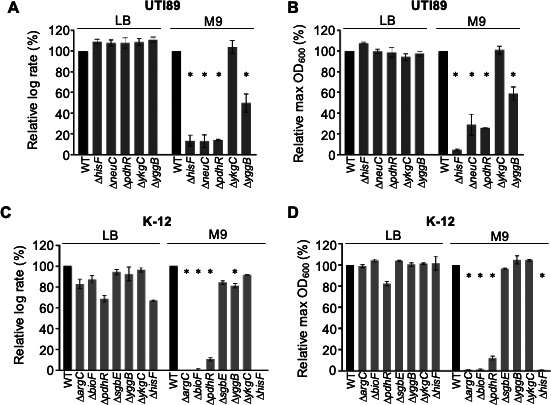
Growth of selected *E. coli* gene deletion mutants. (**A**) The relative log growth rates (µ) determined from growth curves (normalized as a percentage of WT UTI89) grown in LB or M9-glycerol. (**B**) The maximum OD_600nm_ (% of WT) over the 24 h growth period. Panels (**C**) and (**D**) represent the same approach applied to *E. coli* K-12 strain BW25113 and the indicated mutants. Error bars indicate the SD from *n* = 2 independent replicates. *p-value ≤ 0.05 (two-sided *t*-test compared to WT).

We selected five UTI89 genes that showed TraDIS results of differing effect size and statistical strength: *hisF*, *neuC*, *yggB*, *pdhR*, and *ykgC* (Table 2; Table S1E). These five genes were deleted from UTI89 (isogenic parent) using λ-Red recombination, and growth was monitored in M9-glycerol and LB to obtain the mean log-phase growth rates and maximal optical densities (OD_600nm_) as a percentage of wild-type (WT) UTI89 (Fig. 5, with growth curves shown in Fig. S5). In all five mutant strains, growth in LB was indistinguishable from WT. However, after transition to M9-glycerol their responses differed. The deletion of *hisF* resulted in complete inhibition of growth, consistent with the significant difference between M9-glycerol and LB seen in TraDIS (Table 2; Fig. 4C). Deletion of *neuC* or *pdhR* resulted in a strong growth defect in M9-glycerol (Fig. 5A and B). This was expected since the *pdhR* gene is also essential for the growth of *E. coli* K-12 in M9-glycerol (11), although the *pdhR* results had not met the high-stringency thresholds applied to the UTI89 TraDIS data (Table S1E). Deletion of *yggB* in UTI89 resulted in only a moderate growth defect in M9-glycerol and showed a moderate reduction in the TraDIS dataset (LogFC −1.69, *p* = 0.00023). Lastly, deletion of *ykgC* did not affect growth in M9-glycerol (Fig. 5A and B), consistent with the TraDIS screen that showed only a minor fold-change between M9-glycerol and LB that varied between replicates (logFC of −0.64 and −0.11).

We next aimed to determine if some of the UTI89 results above were congruent with findings from a previous screen of *E. coli* K-12 gene knockouts in M9-glycerol (11). We analyzed LB and M9-glycerol growth curves that we performed on seven K-12 strains from the Keio collection of knockout mutants (31). Four of the mutant genes (*hisF*, *yggB*, *pdhR*, and *ykgC*) are the direct homologs of genes analyzed in UTI89 above, whereas the additional three (*argC*, *bioF*, and *sgbE*) were chosen to assess their correspondence with the range statistical strengths that their homologs exhibited in the UTI89 TraDIS analyses (Table 2; Table S1E). Consistent with the UTI89 TraDIS results, the K-12 *argC*, *bioF*, and *hisF* knockout strains grew well in LB but failed to show any significant growth in M9-glycerol (Fig. 5C and D; Fig. S5). The K-12 *pdhR* knockout also grew very poorly in M9-glycerol as expected (11) although, beyond 24 h, eventually grew to high cell densities like the WT (data not shown). The K-12 *yggB*, *ykgC*, and *sgbE* deletions resulted in little or no growth defects in M9-glycerol, consistent with results obtained in UTI89. The growth results for these strains in UTI89 and K-12 therefore show a similar overall profile except for *neuC*, which is not found in K-12 yet has an important role for growth of UTI89 in M9-glycerol. These results support our TraDIS results, and the stringency of the thresholds we applied.

### Validation of select mutants for survival in the human BEC infection cycle analyzed by TraDIS

**Fig 6:**
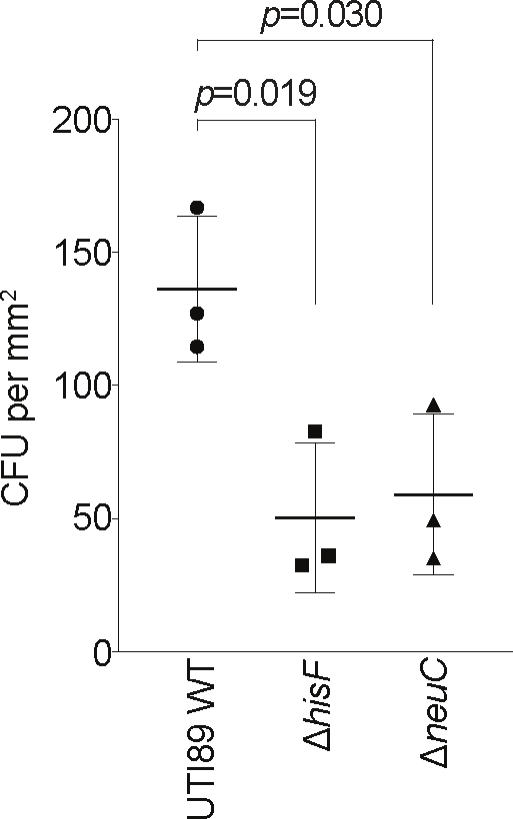
Deletion of *hisF* or *neuC* affects bladder cell infection. Gentamicin-protected bacteria (intracellular) of the indicated strains were recovered at the IBC stage and cfu, per area of the infection surface, were counted. Error bars indicate the SD (*n* = 3). Significance was calculated using a two-sided *t*-test.

Various macromolecule precursor and polysaccharide biosynthetic genes appeared to be important for both the intracellular stage of infection and for growth in M9-glycerol. We chose to independently confirm the importance of the *hisF* and *neuC* genes, which are important in both M9-glycerol and in the intracellular stage of infection (IBC), as implied by TraDIS (Fig. 3 and 4). The *hisF* and *neuC* genes were deleted from UTI89 by λ-Red recombination, and infection experiments were carried out with UTI89 WT, Δ*hisF*, and Δ*neuC* strains. After 24 h of intracellular human BEC growth utilizing a gentamicin protection assay, we observed a significant reduction in bacterial cfu in both the Δ*hisF* and Δ*neuC* strains when compared to the WT (Fig. 6), verifying our TraDIS analyses that both *hisF* and *neuC* are important for intracellular infection of human BECs.

**Fig 7:**
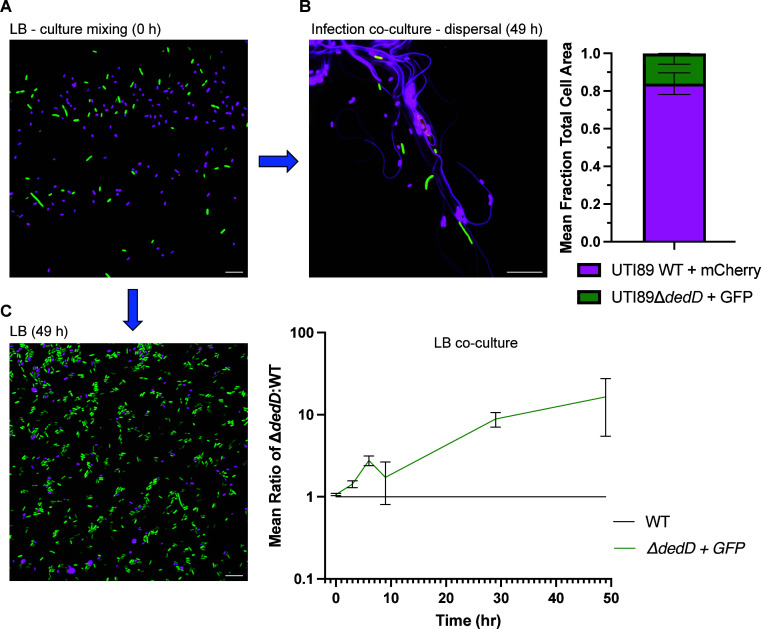
Deletion of *dedD* affects bacterial growth during infection of human BECs. (**A**) Fluorescence composite image of mixed UTI89 WT (isogenic parent; mCherry—magenta) and Δ*dedD* (GFP—green) strains (pre-infection, 0 h). (**B**) The relative biomass (total cell area) was measured after dispersal (20 h urine treatment) from infection (49 h PI; *n* = 3). (**C**) LB co-culture assay, indicating a growth advantage of Δ*dedD* compared to WT; the example image at 49 h PI shows Δ*dedD* (GFP) dominant. Cell area data were normalized at each timepoint using a WT (mCherry) vs WT (GFP) control co-culture, normalized to a ratio of 1 (*n* = 3). Cultures were passaged at 9 h and 29 h (1:1,000 dilution into fresh LB).

To facilitate the further direct testing of the role of genes implicated in infection, we next established a direct competition assay of infection, where UTI89 WT and a mutant were differentially labeled with cytoplasmic fluorescent proteins (GFP or mCherry), and cultures were equally mixed before infection; the proportions of the two strains may be determined at pre- and post-infection stages by fluorescence microscopy. The direct comparison of WT and mutant in the one infection was expected to reduce experimental variance and improve throughput. We applied this method to two genes of interest identified in the dispersal and recovery stage analyzed by TraDIS, *dedD*, and *slmA*, respectively (Table S2), which have roles in regulating cell division. The Δ*dedD* strain grew well against WT in LB co-culture over the same period as used in the infection model (Fig. 7C) but was strongly out-competed in samples taken in the dispersal stage of infection (Fig. 7B), consistent with TraDIS analyses. In separate infection experiments, the Δ*dedD* strain produced filaments visually indistinguishable from WT on dispersal (Fig. S6), further suggesting that *dedD* plays a role in maintaining growth rate or survival during dispersal rather than directly controlling infection-related filamentation (IRF) (5).

SlmA helps prevent cell division from occurring over the DNA during the *E. coli* cell cycle, although a single *slmA* deletion shows a weak phenotype (57). Since DNA forms an irregular extended axial filament within UPEC filaments (8), we hypothesized *slmA* could be especially important for proper division site positioning between DNA as nucleoids condense during filament reversal. However, initial results comparing UTI89 WT and Δ*slmA* recovery in LB culture following BEC dispersal were inconclusive and showed no obvious recovery defect (data not shown). We therefore did time-course imaging to visualize the division of filaments and DNA localization during recovery in LB. This also showed no major defects of Δ*slmA* in DNA condensation into distinct nucleoids or filament division (Fig. S7, Video S1). Further studies will be required to investigate any possible alternative or relatively minor roles of *slmA* at this stage.

## DISCUSSION

To screen for bacterial genes important for infection of host cells in culture, we have developed a modified TraDIS protocol that uses regular Illumina sequencing library prep and is amenable to such analyses where culture-scale and DNA yield may be limited. We applied this method to a transposon-insertion mutant library of *E. coli* UTI89 to identify genes important in the stages of infection of cultured BECs by comparing TraDIS data obtained from each stage of infection to the preceding one. We correlated these data with a TraDIS analyses of the UTI89 transposon mutant library grown in M9-glycerol minimal media. This approach should facilitate comprehensive genome-wide analyses of gene function during stages of other microbial infections or complex developmental pathways.

In characterizing the UTI89 transposon-insertion library, we identified 196 genes meeting a significance threshold that tolerated transposon-insertions during the initial library generation on solid LB but were important for ongoing growth (five overnight passages) in liquid LB culture. Among these were several genes involved in the biosynthesis of the LPS core of the *E. coli* outer membrane, such as *waaY*, *waaL*, and *waaV* (or *rfa* genes), likely reflecting the general importance of outer-membrane stability to cell survival and ongoing growth in culture (58). Some of these genes were also identified in our TraDIS screen for survival during intracellular infection of bladder cells when compared to a pre-infection sample of the library. This might reflect their general importance over time in culture, rather than an infection-specific role. During *in vivo* infection studies, where this may not be practical or possible to do accurately, comparing the screen results to those of batch cultures overtime would therefore help assess the likelihood that identified genes have infection-specific roles.

We also identified 16 genes where transposon-insertions appeared to improve the fitness of UTI89 overtime during passage in LB, some of which may reflect an anticipated limitation due to the scale of the library in our study. Yet others should reflect a genuine enhancement of growth due to gene inactivation, which potentially includes some transcriptional repressors of genes involved in nutrient utilization (e.g., *cytR*; Fig. 1E), like those seen in the original *Salmonella* TraDIS dataset (14). Furthermore, adaptive mutations in *cytR* were found to confer a growth advantage to *E. coli* MG1655 during long-term serial passaging in LB when compared to WT (59). We speculate that these types of genes are dispensable in rich medium, where they could limit maximal nutrient utilization and growth, but would be beneficial for regulating nutrient use in natural environments.

Natural environments like a host cell cytoplasm require certain *de novo* biosynthesis and nutrient utilization functions of infecting bacteria, including during UTI (9, 60–62). Our screens for UTI89 genes required for transition to and growth in M9-glycerol and for intracellular growth in cultured BECs expand on this view and show common types of gene requirements. Many of the strongly identified genes in M9-glycerol and intracellular conditions are implicated in macromolecular precursor biosynthesis, such as amino acids (63), purines (64), and pyrimidines (63, 65), or in the assimilation of nutrients that are consistent with substantial nutrient limitations for bacteria in the host intracellular environment [reviewed in reference (9)]. Independent gene deletion studies in both UTI89 and K-12 strains based on the UTI89 TraDIS results verified the relevance of our screens in the identification of gene functional requirements in M9-glycerol and showed a clear congruence between the specific genetic requirements of both strains, as expected. We verified that the histidine biosynthesis gene, *hisF*, was essential for growth in M9-glycerol and important for intracellular infection of human BECs but was completely dispensable in rich LB medium. This and other bacterial biosynthetic genes strongly identified in our study during infection show the diverse requirements for macromolecular precursor biosynthesis during intracellular infection by UPEC. However, the imperfect correspondence between our analyses and individual tests in some cases highlights the value of independently testing and verifying the role of genes based on TraDIS screen results.

Genes involved in cell envelope stability and stress tolerance were found in both M9-glycerol and intracellular infection screens (Tables 1 and 2). These included outer membrane stabilization factors, such as the Tol-Pal complex, which have been previously implicated in virulence (28). Of particular apparent importance to intracellular infection by UTI89 were genes involved in a range of cell surface polysaccharide biosynthesis, cell envelope stress tolerance, and biofilm-related functions. In our analyses at the 24 h intracellular infection stage, we report at least 10 genes that (to our knowledge) have previously been implicated in UTIs (Table 1), demonstrating the robustness of our approach in identifying biologically real genetic requirements for bladder infection. Interestingly, we found that *neuC*, which is required for the biosynthesis of sialic acid as a precursor for extracellular K1 capsule polysaccharide in some pathogens (16, 66), is important for UTI89 intracellular infection of human BECs. This is consistent with a previous report of K1 capsule involvement in IBC formation in the mouse model of cystitis (67). The role of capsule in UPEC has also been linked to resistance to phagocytosis, immune complement, and survival in blood (16, 68–70). The exact role of *neuC* that improves growth in M9-glycerol is uncertain, but given the importance of other envelope stability and stress-related genes in our data (e.g., *tol-pal* and *rpoS*), this might reflect potential stabilization of the outer cell envelope by the capsule during several of these stress conditions.

The requirement for capsule in various conditions appears to be strain dependent, as, for example, *E. coli* K-12 does not encode the K1 sialic acid capsule but grows well in M9-glycerol (Fig. 3). Both K-12 and UTI89 have another UDP-*N*-acetylglucosamine 2-epimerase distantly related to NeuC called WecB (RffE). However, these proteins are involved in different surface polysaccharide biosynthesis pathways, and the genes are not complementary (16, 66, 71). This is consistent with our observation that *neuC* is important for growth in M9-glycerol despite the presence of *wecB* in UTI89. Overall, these results suggest that K1 capsular polysaccharide has multiple functions, not all of which may be needed in different strain contexts, consistent with the evolving view that gene function is highly strain and context dependent (56).

Our study also searched for genes that function in the latter stages of cellular infection, where bacteria erupt from host cells and disperse into the extracellular environment in differentiated forms, such as highly prevalent filamentous bacteria and motile rods (1). IRF occurs by UPEC cell growth without division in response to multiple conditions encountered during UTI, including urine-specific factors (4, 8, 72). IRF is a likely stress response that may aid in bacterial dispersal (45). The subsequent recovery steps, including filament reversal (division to rods), are thought to be important to allow long-term survival and potential re-infection of other host cells (1, 5). Our observations of envelope integrity and stress response gene requirements for survival of intracellular UPEC, and that some bacteria lyse upon dispersal (5), suggest that envelope stress is a major challenge for bacterial survival during UTI. Consistent with this, genes implicated in cell division and envelope regulation were observed in our dispersal and recovery stage screen results. One of these, *yfiR*, is already implicated in UPEC survival during the mouse model of UTI (55), supporting our TraDIS analyses in the identification of *bona fide* requirements for bladder cell infection. The YfiR protein is thought to be a negative regulator of YfiN (DcgN), which inhibits cell division in response to cell envelope stress (52). Further work will be needed to ascertain the specific mechanistic role during the infection cycle.

One strongly predicted gene in our dispersal screen was *ytfB*. This gene has roles in both cell division (in K-12) and binding to host cell surface glycans in UTI89 (48, 73). A substantial effect of *ytfB* on cell division only became evident when Δ*ytfB* was combined with Δ*dedD* (48), which is thought to play a role in peptidoglycan stabilization during cell division (74). Interestingly, we found that *dedD* has an important role during dispersal from host cells, which was verified by our fluorescence-based competitive infection assay. Since YtfB and DedD both have extracytoplasmic glycan-binding capacities (48, 73, 74), we speculate that these proteins play roles in stabilizing peptidoglycan and cell division or the cell envelope during envelope stress or conditions experienced by UPEC in the latter stages of the infection cycle.

Many genes we identified with potential roles in the dispersal and recovery stages of infection have diverse predicted functions or are currently poorly characterized and warrant future investigation. This includes many genes within the endogenous pUTI89 plasmid including the extrachromosomal *cjr* operon (*cjrABC*) which was previously implicated in the mouse model of UTI (75). We also report the identification of the *fim* operon (*fimACD*) and *fimH* adhesin which has been widely implicated in bladder infection and targeted in UTI therapeutics (76, 77). In summary, the genes identified in our TraDIS analyses represent a spectrum of potential targets for therapeutic intervention in UTI.

## MATERIALS AND METHODS

### Bacterial strains and standard growth conditions

*E. coli* BW25113 wild-type and Keio knock-out strains harboring individual mutants of *hisF*, *argC*, *bioF*, *yggB*, *ykgC*, *sgbE*, and *pdhR* were obtained from the *E. coli* Genetic Stock Center (Yale University, USA). Uropathogenic *E. coli* UTI89 (3) knock-out mutants were constructed using λ-Red recombination (78). Primers containing flanks for Red-recombination and 3′ ends for amplifying the kanamycin cassette from pKD4 are given in Table S1F. Mutants were selected on LB agar supplemented with kanamycin (50 µg/mL) and confirmed using allele-specific PCR with external and internal primers to the kanamycin cassette (primer K1; Table S1F).

Strains were routinely cultured at 37°C on solid or in liquid LB [including 1% (w/vol) NaCl], unless otherwise stated. M9 minimal medium was also used where indicated, including 1% (vol/vol) glycerol as the carbon source (11) and a trace elements solution (79). When culturing the UTI89 transposon mutant library, 50 µg/mL kanamycin was included.

### Construction of a mini-Tn5 transposon mutant library in UTI89

*E. coli* UTI89 was prepared for electroporation as previously described (80). A volume of 50 µL of electrocompetent UTI89 was mixed with 1 µL of EZ-Tn5 <KAN-2> Tn5 transposome mix (Epicenter Biotechnologies) and placed on ice for 5 min in a 2 mm electroporation cuvette. The cells were then electroporated using a BioRad GenePulser, with settings of 2.5 kV, 25 µF, and 200 Ω, and recovered in SOC medium. A total of 11 replicate electroporated samples were then pooled and incubated at 37°C for 1 h. The culture was then concentrated to 1 mL and spread onto LB agar supplemented with kanamycin. The total number of mutant colonies was measured by plate counting of 1/100 and 1/500 dilutions of the main culture (total of 97,500; SEM = 882, *n* = 3). All plates were incubated for 16 h at 37°C. Colonies (lawn) were then resuspended with 1 mL of LB supplemented with kanamycin and 16% (vol/vol) glycerol. This library was stored in aliquots at −80°C.

### Transposon-directed insertion-site sequencing

We used Tn5-transposasome mediated DNA fragmentation coupled to linker attachment (tagmentation) and dual-end transposon insertion-site PCR (Fig. S1) to significantly reduce the amount of input DNA required and eliminate the need for custom sequencing primers and dark cycles during sequencing with the original TraDIS method (14). Genomic DNA (gDNA, 5 ng) from the transposon mutant library (grown as indicated *below* in LB, M9-glycerol, or at distinct stages of bladder cell infection) was subjected to tagmentation using the Nextera DNA Library Prep Kit (Illumina). The DNA was mixed with 10 µL of 2× TD Buffer and 5 µL of Tagment DNA Enzyme TDE1 [diluted 1/50 in 0.5× TE buffer and 50% (vol/vol) glycerol) and made up to a final volume of 25 µL and then incubated at 55°C for 5 min. A volume of 5 µL of 0.2% (w/vol) SDS was mixed with the sample to stop the tagmentation reaction, and the sample was incubated for 5 min at room temperature.

To amplify the transposon-genomic DNA junctions from both transposon ends separately, each PCR combined a transposon-specific outward-directed primer with primers that anneal to one of the end-adapter sequences (Fig. S1C). Both primers include 5′ tag sequences that make them suitable for downstream sequencing (Table S1A), as described *below*. The transposon-specific primer was designed to include (from 5′ to 3′): (i) the “P5” or “P7” flow-cell annealing sequence (Illumina), (ii) a standard “read 1” or “read 2” sequencing primer binding site, and (iii) a ~20 bp transposon-specific annealing sequence, designed to amplify 40–50 bp of the corresponding transposon end followed by its adjacent genomic sequence. Two such transposon-specific primers were designed—one for each end of the transposon; one end was represented by the P7/read one tag, and the other by the P5/read two tag (Fig. S1B). The second primer in each PCR reaction follows the standard design for Illumina sequencing, containing (from 5′ to 3′): (i) the alternate P5 or P7 adapter sequence for binding to the Illumina sequencing flow cell, (ii) a unique barcode for de-multiplexing, and (iii) a sequence that anneals to the tagmentation adapter on the opposing end of the fragment, which then serves as the read primer binding site during sequencing. The dual transposon end sequencing design ensured that only ~50% of reads contained a common transposon sequence during the early cycles of each paired-end read, and the remaining reads comprised genomic sequence reads without transposon sequence (Fig. S1C).

The PCR reactions were carried out using the KAPA HiFi library amplification kit (KAPA Biosciences). Primers (0.2 µM each) were added directly to the tagmentation reaction along with the KAPA HiFi HotStart mix. As a control for non-specific amplification, identical PCR reactions containing a single primer were also performed and did not generate a product (data not shown). An initial extension step was performed at 72°C for 3 min. This was followed by a denaturation step at 98°C for 30 s, followed by 28 cycles of: 98°C for 15 s, 55°C for 30 s, and 72°C for 30 s. A final extension at 72°C for 5 min was performed.

The purified PCR products were subjected to standard Illumina paired-end sequencing (Fig. S1C). Multiplex sequencing with samples from both transposon ends together provided the necessary read diversity during Illumina sequencing, avoiding the need for custom dark cycles. The amplified DNA samples were quantified with an Agilent Bioanalyzer and pooled to equimolar concentrations and underwent SPRI-select magnetic bead clean-up (Beckman-Coulter) at 0.9×–0.5× left and right ratios, respectively (to select DNA of 200–800 bp). PhiX genomic adapter-ligated DNA library (Illumina) was included at 5% of the total DNA. DNA was then denatured, and 10 pM was sequenced on a V3 paired-end Illumina flow cell using a MiSeq sequencer for 300 cycles (2 × 150) for M9-glycerol or on an Illumina HiSeq 2500 sequencer for 250 cycles (2 × 125) for the infection samples (UTS Sequencing Facility, NSW Australia).

### Analysis of TraDIS data

Sequence reads from the multiplex fastq files were separated into individual files, according to their unique index barcodes. The data underwent quality control filtering using FastQC (v0.11.2; Simon Andrew, 2010, http://www.bioinformatics.babraham.ac.uk/projects/fastqc/). Sequence reads containing a match to the designated i5 and i7 transposon ends were isolated, allowing up to three mismatches (Table S1A), trimmed off the transposon sequence, and then mapped to the UTI89 chromosome (Table S1G) and pUTI89 (Table S1H) using the SMALT short read mapper, allowing no mismatches, as described previously (81).

The number of mapped reads in bins along the genome was determined with deepTools2 (82). The number of transposon-insertion sites for each gene and the comparative analyses between two conditions were done using Bio-TraDIS (81); the two read files for both transposon ends were input separately for each biological replicate. Genes that contained ≤5 reads were removed from the comparison using EdgeR. Genes meeting the defined statistical thresholds (*p* ≤ 0.05 and logFC ≤ −1.0) in both replicate cultures were identified (Tables 1 and 2); the fold-change and significance scores (*p*-value and *q*-value—the false discovery rate) were given as the means from the two independently analyzed biological replicates.

### Culturing the UTI89 library in LB and M9-glycerol for TraDIS

To carry out five sequential passages of the UTI89 mutant library in LB, an aliquot of the UTI89 transposon mutant library was first thawed on ice, and a sample was taken for DNA extraction using an Isolate II Genomic DNA purification kit (Bioline). Also, 50 µL of the thawed library aliquot was used to inoculate 20 mL of LB, and this culture was incubated for 24 h at 37°C with 180 rpm shaking (passage 1). A volume of 50 µL of the passage 1 culture was then used to inoculate 20 mL of LB and incubated the same way for a further 24 h (passage 2). This was sequentially repeated for a total of five consecutive passages. After each passage, 10 mL of each culture was harvested for genomic DNA extraction and purification as *above*.

To compare the growth and survival of the UTI89 transposon-insertion mutants in M9-glycerol and LB, an aliquot of the mutant library was thawed and diluted in LB (to 150,000 cfu/mL), which was then incubated at 37°C for 1.5 h. One milliliter from the LB starter culture was then added to 9 mL LB, and another 1 mL of the starter culture was added to 9 mL M9-glycerol. These cultures were incubated for 24 h at 37°C with 200 rpm shaking. A volume of 1 mL from each culture was then used to inoculate 10 mL of the corresponding fresh medium and grown for another 24 h at 37°C with 200 rpm shaking. Finally, 10 mL from this culture was then used to inoculate a final volume of 90 mL of fresh medium and grown for 24 h at 37°C with 200 rpm shaking. Samples were harvested for genomic DNA extraction and TraDIS. This sequential dilution from LB into M9-glycerol supported the transition between media and supplied sufficient auxotrophic requirements for UTI89 growth (83).

### Culture of individual deletion mutants in M9-glycerol and LB

Single colonies of wild-type and knockout mutant strains were used to inoculate 5 mL of pre-warmed liquid LB (supplemented with kanamycin, where appropriate), and the cultures were incubated at 37°C with shaking for 16 h. A volume of 500 µL was then used to inoculate 5 mL of LB or M9-glycerol and incubated at 37°C with shaking for 16 h. Cultures were used to inoculate 200 µL of fresh LB or M9-glycerol in 96-well sterile microtiter plates to give a starting OD_600nm_ = 0.015. The plate was incubated at 37°C for 24 h with low shaking and analyzed with a microtiter plate spectrophotometer (BioTek), measuring the OD_600nm_ at 30 min intervals. The growth curves were obtained by averaging technical replicates within two independently performed microtiter plate experiments. The DMFit (DM: Dynamic Modeling, version 3.5) growth curve modeling software (84) was used to obtain values for the lag, growth rate (μ), and maximum OD.

### Infection of bladder epithelial cells with the UTI89 library for TraDIS

Human bladder cell line PD07i was cultured at 37°C with 5% CO2 to confluency (4, 8). Bladder cells were trypsinized and concentrated to 4 × 10^5^/mL, and 10 mL was dispensed into each sterile tissue culture dish (BD Falcon, 100 mm diameter × 20 mm depth) and incubated at 37°C with 5% CO_2_ for 24 h. For TraDIS analyses, each two biological sample replicates harvested from the distinct stages of infection below were made up of three pooled replicates (*n* = 6).

#### IBC stage of infection

An aliquot of the UTI89 transposon mutant library was thawed, and 100 µL was used to inoculate 10 mL of liquid LB and grown statically overnight at 37°C. The bacterial culture was then diluted to OD_600nm_ = 1.0 in PBS, and 1 mL of the dilution was used to inoculate each bladder cell culture dish (MOI = 100). The culture dishes were then centrifuged for 5 min at 2,000 rpm and left to incubate statically for 2 h at 37°C with 5% CO_2_. The supernatant was then discarded, and 10 mL pre-warmed EpiLife medium (Thermo-Fisher) supplemented with human keratinocyte growth supplement (HKGS) and gentamicin (100 µg/mL) was added. Culture dishes were then incubated statically for 22 h at 37°C with 5% CO_2_. Culture dishes were imaged in phase-contrast and GFP fluorescence (UTI89 pGI5) (4) at 18 and 22 h using a Nikon Ti inverted microscope which confirmed dense biofilm-like IBCs. After 22 h of static growth, the supernatant was removed, and the culture was gently washed four times with PBS. Lysis solution (0.5% trypsin-EDTA and 0.1% triton X-100) was added and incubated for 10 min at 37°C. The entire supernatant was then collected and spun at 4,600 rpm for 10 min. The supernatant was removed, and the cell pellet underwent gDNA extraction and purification.

#### Host cell dispersal stage of infection

To induce bladder cell dispersal, the infected bladder cells were exposed to human urine (4, 5). Urine was centrifuged at 4,600 rpm for 10 min, and the supernatant was filter sterilized (with a 0.2 µm filter). Filtered urine samples were then pooled (density = 1.026 g/mL) and stored in 40 mL aliquots at −20°C. Each aliquot was thawed at 37°C before use. Following 22 h of intracellular infection, as described *above*, the Epilife medium was removed, and the dishes were washed four times with PBS. Ten milliliter of urine was added, and the culture dish was left to incubate at 37°C with 5% CO_2_ and gentle orbital shaking (~50 rpm to resuspend bacteria released from the bladder cells). During incubation, 10 mL of overlay urine was periodically withdrawn for analysis and immediately exchanged for fresh 10 mL of pre-warmed urine, after incubation for 20 min windows over 10 h. The short windows of collection aimed to minimize recovery and growth of the mutants in the urine (Fig. 4C). The collected samples were centrifuged at 4,600 rpm, and the pellets were frozen before undergoing gDNA extraction and purification.

#### Bacterial recovery from infection

From the 10 h time point of urine exposure (34 h PI), urine was collected in 2 h collection windows (pre-warmed urine was re-applied after each collection), centrifuged, and stored on ice. Once all samples were collected, the cell pellets were resuspended in 10 mL of warm liquid LB. Cultures were pooled and then incubated for 18 h overnight at 37°C with 180 rpm shaking. Twenty milliliter of this culture was centrifuged at 4,600 rpm for 10 min, and the cell pellet underwent gDNA extraction. LB was chosen as the recovery media to avoid further selection pressure and allowed a direct comparison to the serial 5-day LB passaging experiments. A further 1/500 dilution into liquid LB of the 18 h recovered culture was done, and the culture was incubated for a further 24 h with 180 rpm shaking. This aimed to add an additional selection step for identifying mutants that had recovered. Following the extra 24 h growth, the culture was centrifuged at 4,600 rpm for 10 min, and the cell pellet underwent gDNA extraction and purification.

### Infection assay of *hisF* and *neuC* deletion strains

Human bladder cell line PD07i was cultured at 37°C with 5% CO2 to confluency as described *above* and counted using a Coulter Counter (Beckman) to a minimum of 1 × 10^5^ cells/mL. A 0.5% Trypsin-EDTA mixture and defined trypsin inhibitor (Gibco) were used to release bladder cells from the base of flasks. Bladder cells were concentrated to 4 × 10^5^/mL, and 1 mL was dispensed into a six-well sterile culture dish (BD Falcon; 35 × 18 mm^2^) and incubated at 37°C with 5% CO_2_ for 24 h. Bacterial cultures for UTI89 WT (isogenic parent), Δ*hisF*, and Δ*neuC* strains were grown overnight statically and then diluted down to an OD_600nm_ = 1.0. This was used to inoculate the bladder cells by adding 0.1 µL/mm^2^ of bacteria (MOI = 100). The plates were then spun down for 5 min at 2,000 rpm and incubated statically for 2 h at 37°C with 5% CO_2_. The supernatant was discarded, and bladder cells resuspended in EpiLife supplemented with gentamicin (100 µg/mL) and HKGS, as *above*. Cultures were incubated statically at 37°C for 22 h. Following this, culture dishes were washed gently with PBS four times, and lysis solution (0.5% trypsin-EDTA and 0.1% triton X-100) was added and incubated for 10 min at 37°C. The entire supernatant was then collected and plated onto LB agar and left to incubate at 37°C for 16 h. Colonies were then counted from a 1/100-dilution series. This experiment and the results presented in Fig. 6 was performed using three replicates (*n* = 3).

### Growth competition (co-culture) assays

Wild-type UTI89 and each gene deletion mutant strain were transformed with pGI5 (GFP) or pGI6 (mCherry) (4). A single colony of each strain was used to inoculate LB medium, supplemented with 100 µg/mL spectinomycin, and the cultures were incubated at 37°C. Each culture was then diluted to an OD_600nm_ = 0.20 and mixed equally. This starting mixture was imaged by fluorescence microscopy (0 h sample).

For co-infection assays, a sample of an equally mixed bacterial culture was used to infect confluent PD07i bladder epithelial cells in flow chambers, as described previously (5). In the Δ*dedD* experiment, we omitted the non-essential flushing step (100 µL/min) at the start of infection. For the reference LB co-culture assays, we passaged and sampled the co-culture at times corresponding to the main stages of the infection model (4). The mixed bacteria were diluted in LB (OD_600nm_ = 0.010) and incubated at 37°C with 200 rpm shaking and sampled at 3, 6, and 9 h for microscopy. At 9 h, the culture was diluted 1/1,000 and after a further 20 h of growth (i.e., 29 h total), sampling for microscopy and culture dilution (1/1,000) were carried out again. The diluted culture was incubated as above for a further 20 h before for imaging (49 h sample). The total area of cells of each strain in fluorescence micrographs was used as a measurement of the relative biomass. At each timepoint, the data for a control co-culture (WT-mCherry vs WT-GFP) were normalized to 50% each (i.e., a ratio of 1). This normalization factor was then applied to the WT-mCh vs Δ*dedD*-GFP co-culture sample for each timepoint.

### Microscopy

Bacterial samples (1–3 μL) were placed onto 1%–1.2% (w/vol) agarose pads (in PBS or LB) on a glass slide, and a cover slip was placed on top. Bright field and fluorescence imaging were performed using a Nikon Ti2-E microscope equipped with a 100 × 1.4 NA objective and GFP or mCherry filter sets where appropriate. All image analyses was conducted in FIJI to determine the total fluorescence area of cells of each strain, representing biomass. Statistical analyses and data representation were done using GraphPad Prism 9.2.0.

## Data Availability

Nucleotide sequence data have been deposited under NCBI BioProject ID PRJNA1047296.

## SUPPLEMENTAL MATERIAL

Supplemental figures msystems.00387-24-s0001.pdf Figures S1 to S7.

Table S1 msystems.00387-24-s0002.xlsx Construction and characterization of the UTI89 transposon-insertion mutant library by TraDIS analyses.

Table S2 msystems.00387-24-s0003.xlsx Profiling the UTI89 genes required for bladder cell infection.

Movie S1 msystems.00387-24-s0004.avi Time-lapse imaging of filament reversal (division) on LB agar.

ASM does not own the copyrights to Supplemental Material that may be linked to, or accessed through, an article. The authors have granted ASM a non-exclusive, world-wide license to publish the Supplemental Material files. Please contact the corresponding author directly for reuse.
