# Supplementary material for: Genetic requirements for uropathogenic E. coli proliferation in the bladder cell infection cycle
Source: mSystems. 2024 Sep 17;9(10):e00387-24. doi: 10.1128/msystems.00387-24 (PMC11495030; doi:10.1128/msystems.00387-24)
Supplement: Supplemental figures — Figures S1 to S7. [file msystems.00387-24-s0001.pdf]

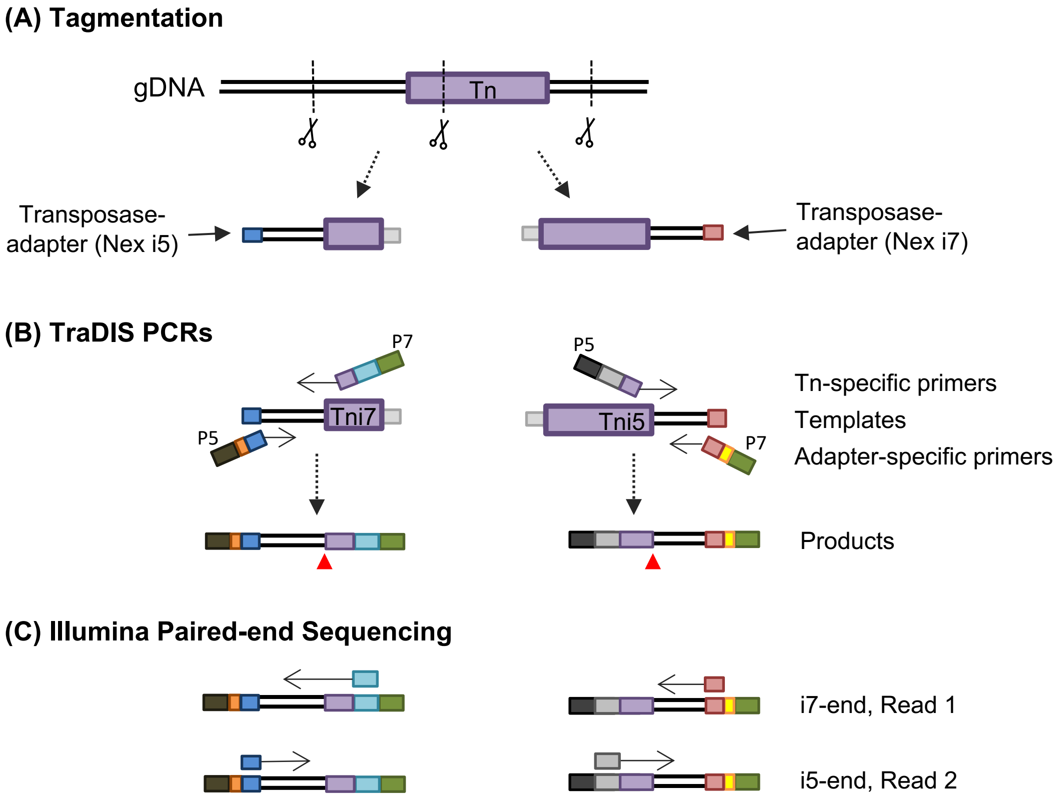

Supplementary Figure 1. Outline of the modified transposon-directed insertion-site sequencing (TraDIS) protocol. (A) A genomic region containing a transposon (Tn) insertion site from the library is represented here. Genomic DNA (gDNA) is first randomly fragmented via tagmentation. In this reaction, the transposase cuts dsDNA and attaches standard Nextera-Illumina adapters (Nex i5, blue, or Nex i7, red) to the ends of the fragmented DNA. **(B)** Each end of the transposon is designated ("Tni5" and "Tni7") by respective Tn-specific outward-directed primers that incorporate the appropriate 5' Illumina flow-cell adapter (P5 or P7; dark grey and dark green, respectively) and read primer binding site (read 1 – cyan, and read 2 – light grey). For PCR, the opposing primer anneals to the Nex i5 or Nex i7 adapters (blue and red, respectively). These primers also contain unique barcodes to identify each sample (orange and yellow), and the alternate flow cell binding sequences (dark grey or dark green). The TraDIS PCR amplifies the transposon-gDNA junctions (indicated by red arrow-heads in the PCR products). **(C)** Paired-end sequencing is then carried out with standard Illumina workflows; multiplex sequencing of samples amplified from both ends of the transposon ensures high sequence diversity during both read 1 and read 2. For data analysis, high-quality sequence reads containing the expected transposon end sequence are selected, precisely identifying the collection of insertion sites.

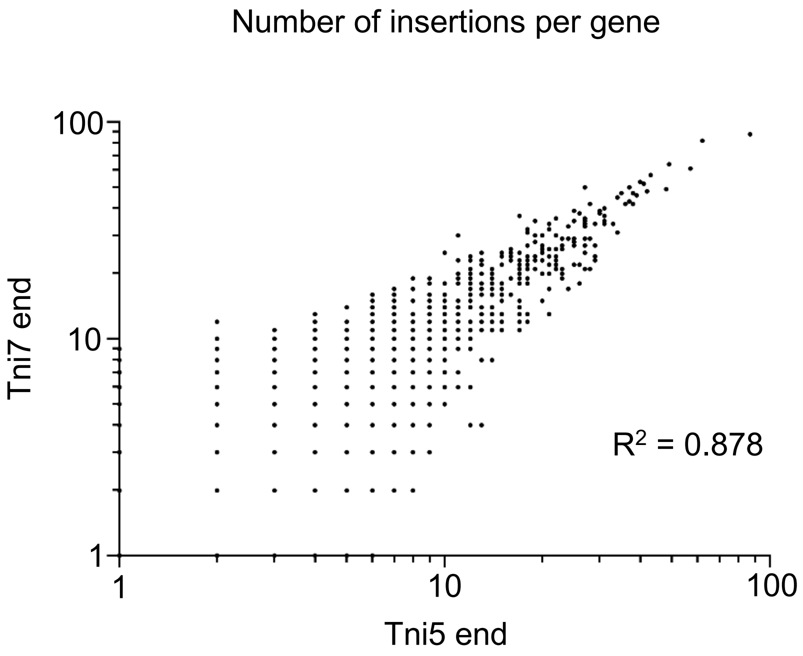

Supplementary Data Figure 2. Correlation between the Tni5 and Tni7 sequencing ends of the *E. coli* UTI89 mini-Tn5 library. The number of insertion sites per gene in UTI89 was determined by sequencing the Tn-gDNA junctions from both the designated "Tni5" and "Tni7" ends of the transposon, mapping these reads the the UTI89 genome, and then identifying the number of specific insertion sites for each gene. For each gene, the number of insertions detected from the Tni5 and Tni7 ends was plotted; note that most points represent many genes.

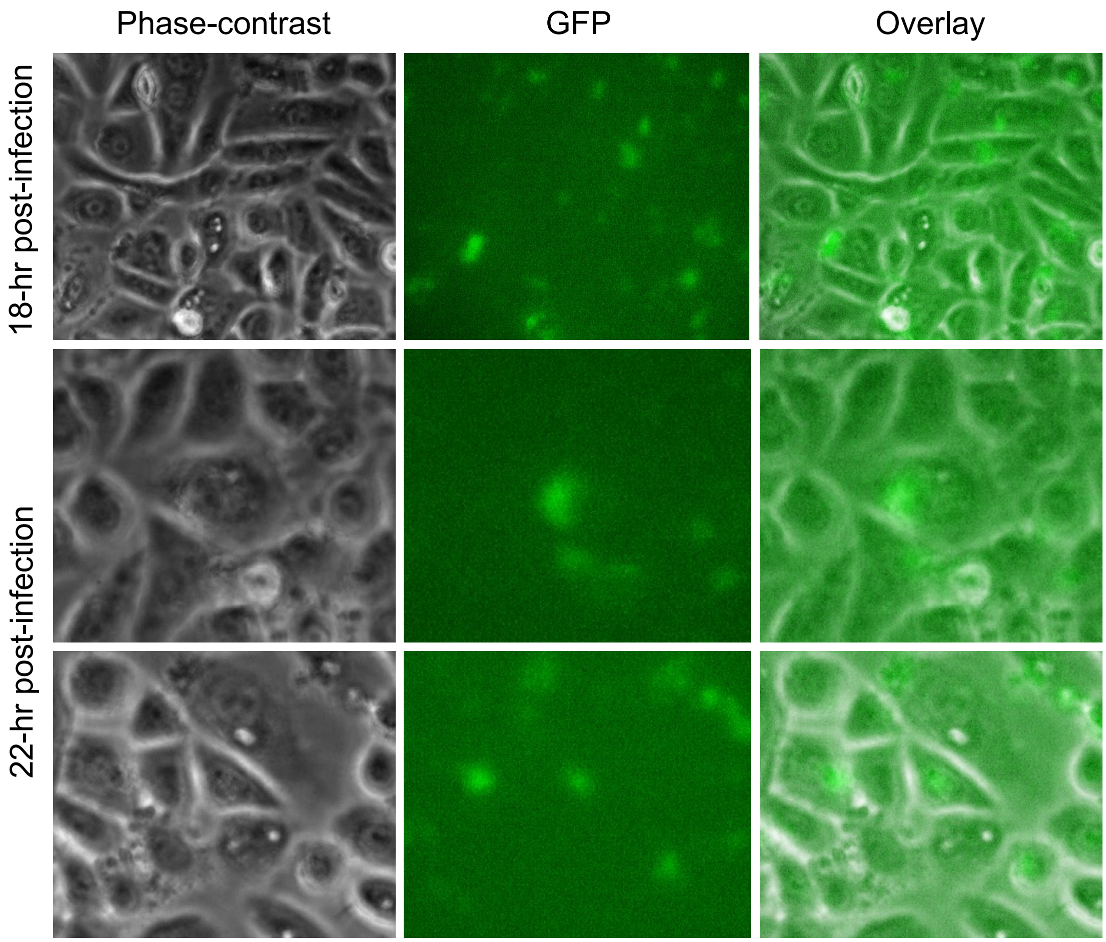

Supplementary Data Figure 3. Human cultured BEC infection model permits UTI89 IBC development. UTI89 pGI5 (wild-type with GFP constitutively expressed) formed dense IBCs which represented previously described IBCs, albeit with lower resolution due to the limitations imposed by the culture dishes. PD07i bladder cells grown in 100 x 20 mm culture dishes were inoculated after static growth in liquid LB and allowed to grow for 2 h. Gentamicin containing media was applied and culture dishes were incubated for a further 22 h to induce intracellular bladder cell growth. Bladder cells were imaged using GFP at 18 and 22 h post-infection.

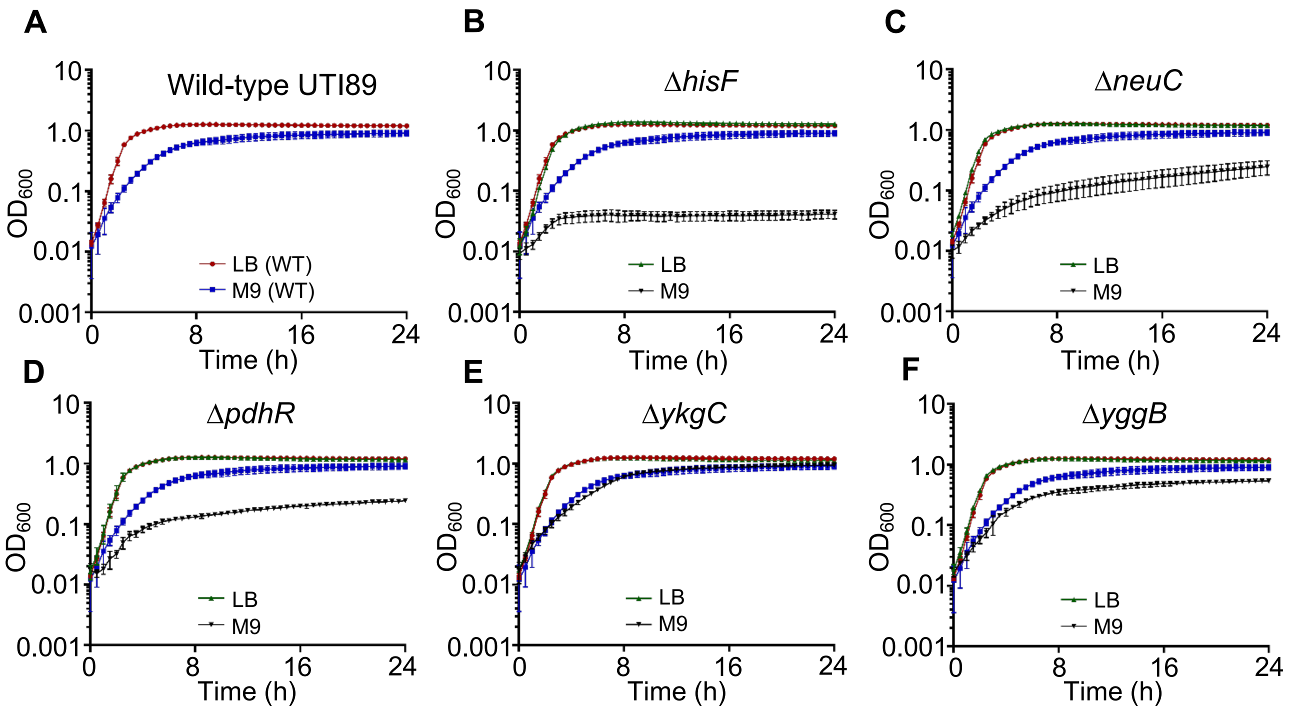

Supplementary Data Figure 4. Growth curves of selected *E. coli* UTI89 gene deletion mutants. Wild-type UTI89 and the indicated deletion mutants grown in LB or M9-glycerol, as indicated. The wild-type UTI89 growth curves in LB (red) and M9-glycerol (blue) from (A) are also shown in these colors on the graphs for the deletions (B-F) for comparison.

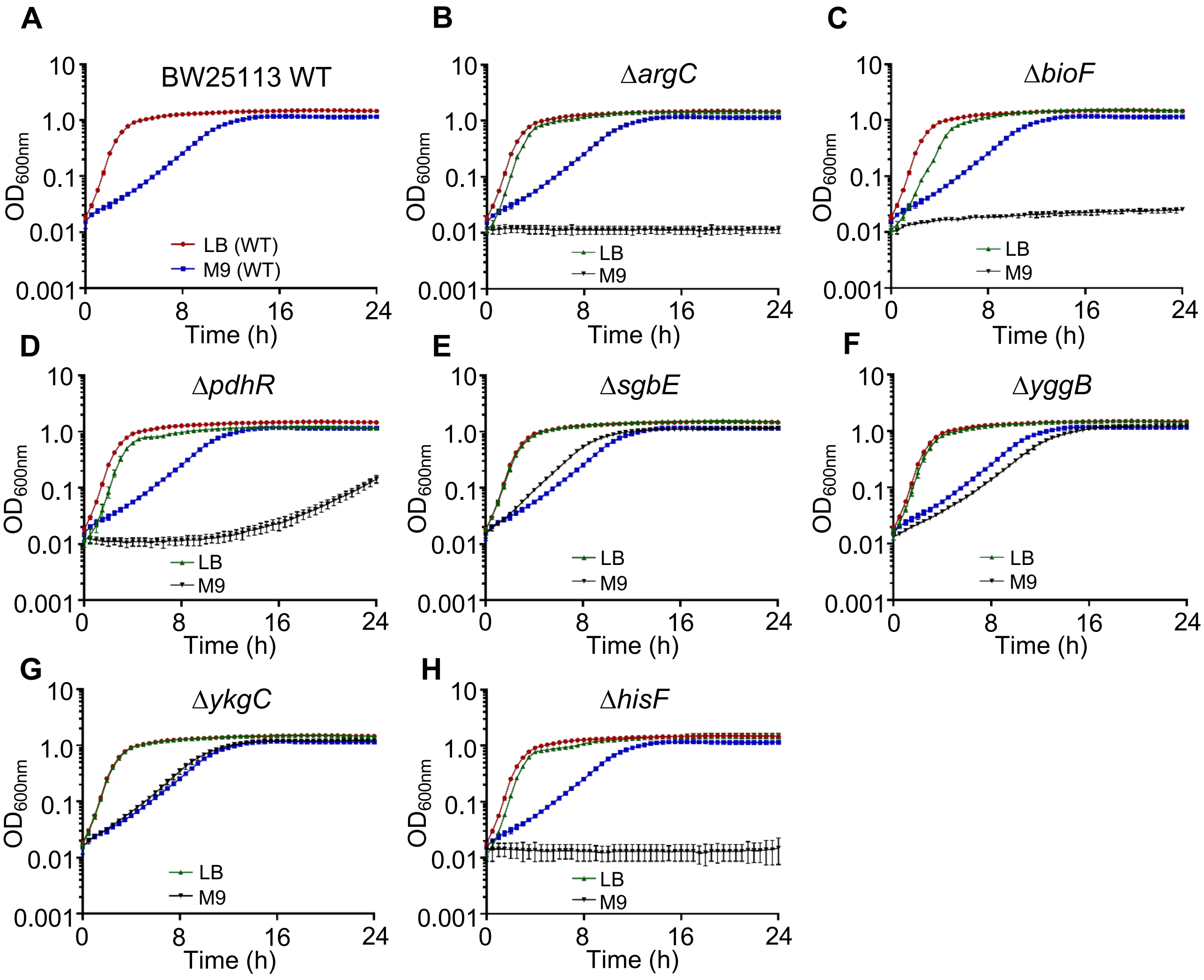

Supplementary Data Figure 5. Growth curves of selected *E. coli* K-12 (BW25113) gene deletion mutants (from the KEIO collection). (A) BW25113 wild-type and the indicated deletion mutants grown in LB or M9-glycerol, as indicated. The wild-type BW25113 growth curves in LB (red) and M9-glycerol (blue) from (A) are also shown in these colors (B-H) for comparison.

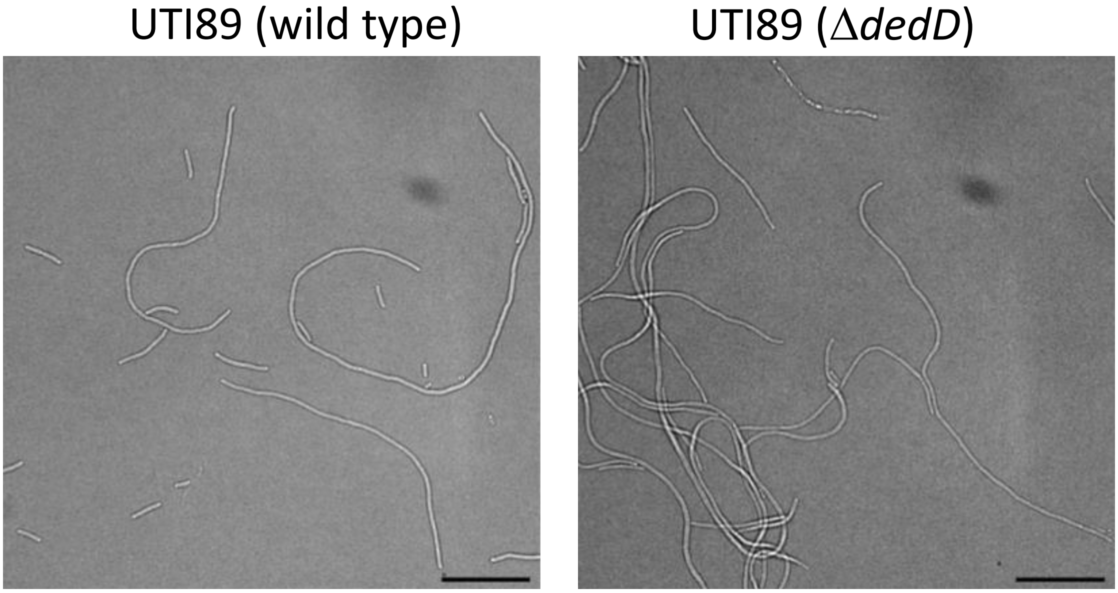

Supplementary Data Figure 6. *dedD* deletion in UTI89 does not substantially affect infection-related filamentation (IRF) during dispersal from infection. UTI89 wild-type and Δ*dedD* strains were sampled at dispersal stage of infection and then imaged by brightfield microscopy. Scale bars, 20 μm.

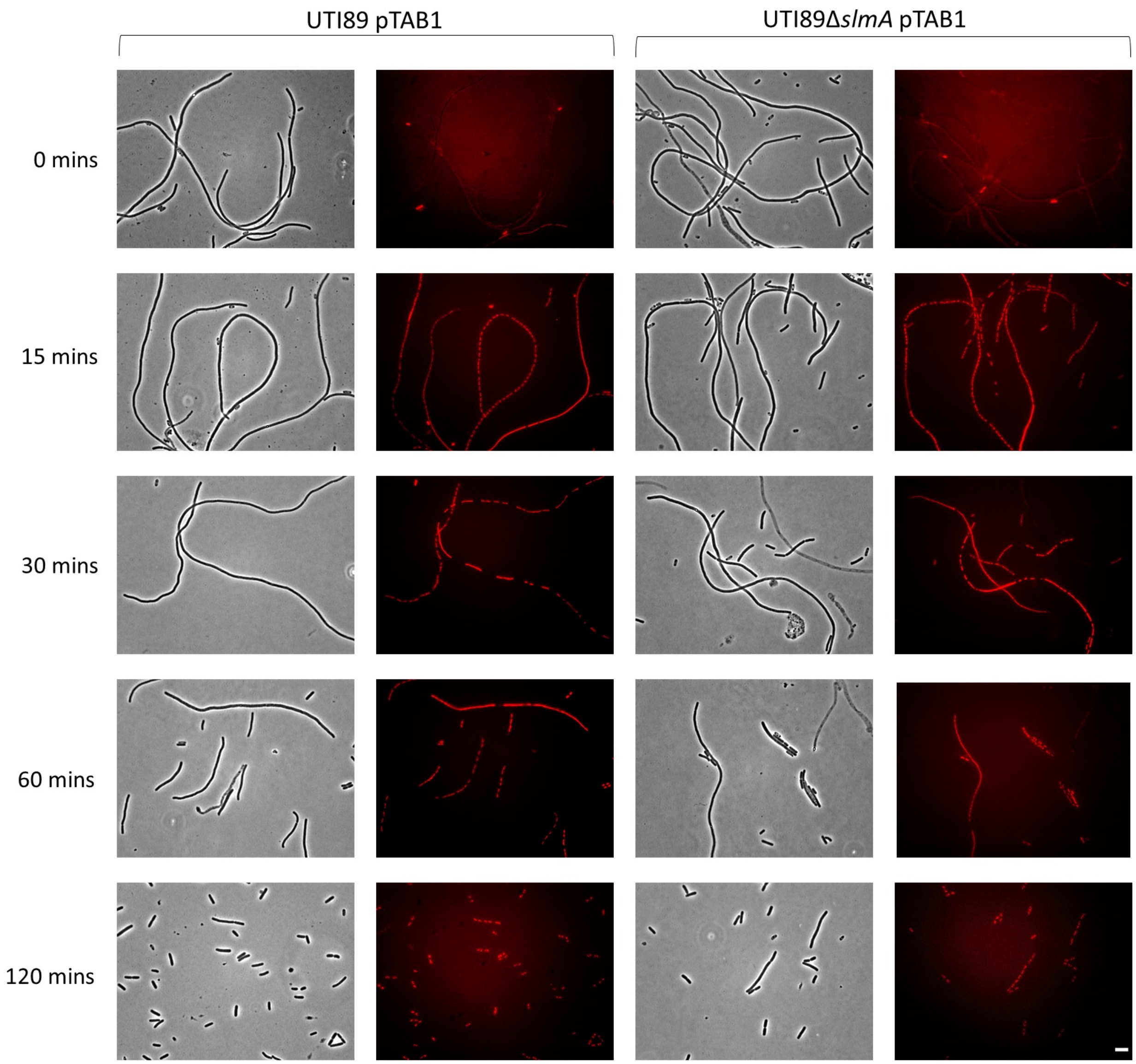

Supplementary Data Figure 7. *slmA* deletion in UTI89 does not substantially affect filament reversal during recovery from infection. Wild-type and UTI89. Δ*slmA* strains (containing an empty vector, pTAB1) were collected at dispersal stage of infections, and then grown in liquid LB culture and sampled at the indicated times for phase contrast (left) and fluorescence (right) microscopy – cells were fixed on sampling with 4% paraformaldehyde and then stained with 1 μg/mL DAPI DNA stain prior to microscopy. By 120 min, filaments of both strains had undergone substantial reversal into shorter rods. Scale bar, 5 μm (applicable to all images). Similar results were seen with time-lapse imaging of filament reversal on LB agar in situ (Supplementary Movie 1).
